# Refractory Hypotension: A Detailed Case Discussion and Current Literature Review

**DOI:** 10.7759/cureus.29558

**Published:** 2022-09-25

**Authors:** Mohammad B Memon, Nader Chadda, Ali Dahhan, Marek Krystofiak, Shaival Thakore

**Affiliations:** 1 Graduate Medical Education, HCA Florida Bayonet Point Hospital, Hudson, USA; 2 Cardiology, HCA Florida Bayonet Point Hospital, Hudson, USA; 3 Cardiology, University of South Florida Morsani Graduate Medical Education/HCA Florida Bayonet Point Hospital, Hudson, USA; 4 Internal Medicine, HCA Florida Bayonet Point Hospital, Hudson, USA; 5 Internal Medicine, University of South Florida/HCA Florida Bayonet Point Hospital, Hudson, USA

**Keywords:** hypotension treatment, human physiology, hyperadrenergic orthostatic hypotension, postural orthostatic tachycardia syndrome diagnosis, neurogenic orthostatic hypotension

## Abstract

Orthostatic hypotension (OH) commonly affects the elderly and can be challenging to manage. Its symptomology can be variable and is induced by decreased cerebral perfusion. Here, we present a complex yet compelling case of a patient with refractory OH, focusing on delineating current medical therapies and pathophysiology for in-training medical professionals.

## Introduction

Syncopal or pre-syncopal episodes caused by orthostatic hypotension (OH) can lead to multiple clinic or emergency department visits. While not restricted to a defined age range, OH is a frequent pathology in the elderly and is of particular concern given the potential consequences of symptomology, such as trauma from falls. Patients usually experience transient loss of consciousness during positive orthostatic episodes due to decreased cerebral perfusion.

Following collaboration among multiple international entities involved in the formulation of medical guidelines, unified criteria for diagnosing OH were established and defined as a reduction of 20 mmHg systolic blood pressure (SBP), or 10 mmHg in diastolic blood pressure (DBP), in three minutes of standing or against gravity on a tilt-table (head up, at least 60) [1]. A modified definition for patients with underlying hypertension defines OH as a decrease in SBP of ≥30 mmHg. Here, we present the case of a patient with refractory OH using the management challenges to review the pathophysiology and discuss available treatments. Our goal is to assist in-training medical professionals in the management of OH.

## Case presentation

An 82-year-old male patient with a medical history of chronic obstructive pulmonary disease (COPD), not on supplemental oxygen, hypothyroidism, right internal carotid artery stenosis on dual antiplatelet therapy, bladder cancer status post-chemotherapy, and benign prosthetic hyperplasia (BPH) presented to the emergency department (ED) after syncopal episodes and suffering a ground-level fall. On admission, the patient complained of dizziness and headache. Before the trauma, the patient reported multiple syncopal episodes in the past two weeks initiated by standing from a seated position.

The patient’s physical examination was remarkable for a superficial forehead laceration. In the ED, the patient’s vitals were a temperature of 98°F, heart rate (HR) of 66 beats/minute, respiratory rate of 16 breaths/minute, blood pressure (BP) of 115/65, and SpO_2_ of 98% on room air. Initial complete blood count was remarkable for hemoglobin of 11.1 g/dL (13.7-17.5), mean corpuscular volume of 93.6 fL (79.0-92.0), and platelets of 101,000 µL (150,000-450,000). The patient’s complete metabolic panel and coagulation panel were within normal limits. Computed tomography (CT) of the head without contrast was remarkable for right frontal subarachnoid hemorrhage (SAH). Neurosurgery recommended no surgical intervention.

On day 2, cardiology was consulted for episodes of bradycardia and syncope preceding and during admission. Medical records were reviewed, and BPH alpha(1)-outflow antagonist medication, tamsulosin, was held due to concerns for iatrogenic hypotension. Orthostatic vials were obtained (Table 1).

**Table 1:** Orthostatic vitals. BP: blood pressure; HR: heart rate; SBP: systolic blood pressure; DBP: diastolic blood pressure; BPM: beats per minute

Position	BP (SBP/DBP)	HR
Laying down	101/55 mmHg	62 BPM
Sitting	113/53 mmHg	72 BPM
Standing up	84/45 mmHg	74 BPM

Carotid Dopplers revealed stable carotid disease, similar to a prior study two years ago. Clinically, the patient appeared to be hypovolemic, and a bolus of 1 L of normal saline (NS) was administered. After completing the bolus, the patient was hemodynamically stable, with SBP ranging from 105 to 125 mmHg and DBP ranging from 71 to 83 mmHg. Repeat orthostatic vitals were also positive. A transthoracic echocardiogram (TTE) revealed an ejection fraction of 55%, grade 1 diastolic dysfunction, with mild mitral valve and tricuspid valve regurgitation.

Electrocardiogram (EKG) showed sinus bradycardia with first-degree atrioventricular block. The patient was started on 5 mg midodrine three times a day (t.i.d.). On day three, the patient had a repeat OH episode, and midodrine was increased to 10 mg t.i.d. The tilt-table test was deferred as the etiology of the syncopal episode was attributed to OH.

On day five, the patient was hemodynamically stable with SBP averaging around 115-125 mmHg on midodrine 10 mg t.i.d. On day six, the patient attempted to ambulate with physical therapy and had another OH episode. Fludrocortisone 0.1 mg was added to the patient’s regimen. He was also started on maintenance fluids at 75 cc/hour NS and thromboembolism-deterrent (TED) hose-knee high stockings to his bilateral lower extremities.

On day seven, the patient continued to have persistent OH with ambulation even though his baseline BP improved on medical therapy. The electrophysiology (EP) service was consulted who agreed with midodrine 10 mg t.i.d. and recommended increasing the fludrocortisone dose to 0.2 mg.

A hormonal lab workup consisting of thyroid-stimulating hormone (TSH), free thyroxine (free T4), triiodothyronine (T3), and morning cortisol was unremarkable. Cortisol was normal at 14.8 µg/dL (6-23). TSH was normal at 2.93 µIU/mL (0.36-3.74), and free T4 and T3 were within normal limits (Table 2).

**Table 2:** Thyroid and cortisol lab findings. TSH: thyroid-stimulating hormone; Free T4: free thyroxine; T3: triiodothyronine

Lab	Normal range	Day 7
TSH	0.36–3.74	2.93 mIU/mL
Free T4	0.7–1.8	1.67 ng/dL
T3	0.9–2.8	2.3 nmol/L
Morning cortisol	6–23	14.8 µg/dL

The patient’s scheduled breathing treatment of ipratropium bromide/albuterol with budesonide was increased to every four hours (q4hr) from six hours (q6hr) in hopes positive inotropic and chronotropic effects might mitigate OH symptomology. On day eight, the patient had repeat episodes of OH upon standing but had supine hypertension, with BP notably 170/98 mmHg. Midodrine and fludrocortisone were temporarily held, but the BP dropped to 86/62 mmHg. One liter NS bolus was given with 100 cc/hour maintenance fluids until a total of 4 L were given over the next 48 hours. On day 10, the patient had an unrelated gastrointestinal discomfort due to small-bowel obstruction, which resolved after the placement of a nasogastric tube.

On day 11, physical therapy re-attempted to ambulate the patient, but the patient suffered another OH episode. Midodrine and fludrocortisone were resumed, but breathing treatments were changed back to q6hr. On day 13, the patient continued to have OH and was started on Droxidopa 100 mg t.i.d., along with the continuation of midodrine and fludrocortisone. On day 15, the patient was evaluated by a rehabilitation service, and on day 17 was discharged to an inpatient rehabilitation center.

Two weeks after discharge to inpatient rehabilitation, the patient suffered another ground-level fall with similar OH symptoms. The trauma team re-evaluated him in the ED and appreciated no changes to his underlying SAH. Once stable, he was transferred to another rehabilitation facility.

## Discussion

Pathogenesis

According to Ricci et al., OH usually affects the elderly and patients with prior neurodegenerative diseases, hypertension, or diabetes [2]. Cardiovascular hemostasis depends on synchronous collaboration by multiple organ systems, such as the cardiovascular, renal, and neural systems. The autonomic nervous system (ANS) consists of the sympathetic nervous system (SNS) and the parasympathetic nervous system (PNS). BP is regulated by the sympathetic branch of the ANS, which modulates the distribution of CO and BP at the arterial level. In contrast, the PNS affects BP through negative inotropic and chronotropic effects [2].

When a patient stands up, gravity causes venous pooling. Nearly 500-1,000 mL of blood is pooled into gravity-dependent lower extremities and the splanchnic veins [3-5]. According to Thompson et al., standing upright and standing causes nearly 11% of total plasma volume pools into the lower extremities [3]. Pooling leads to decreased venous circulation→decreased preload→decreased SV→decreased CO→and decreased BP, which eventually leads to decreased cerebral perfusion. Compensatory mechanisms are activated when baroreceptors trigger a multiorgan response. The respiratory system also aids in hemostasis by increasing intra-abdominal pressure, restricting retrograde blood flow back to the iliac and femoral veins. This increases preload, which subsequently increases CO [4]. Failure of such responses leads to patients having syncopal and presyncope episodes [5]. OH can then be broken down into three types, as described by Brignole et al., namely, classic, initial, or delayed (progressive) [6]. (1) Classic: failure to increase total peripheral resistance (TPR) and HR due to ANS failure. (2) Initial: TPR decreased while CO increased. (3) Delayed or progressive: decrease venous return (reduced preload), but pathogenesis unknown.

According to Brignole et al., risk factors for OH include older age, periods of persistent standing, exposure to increased temperatures, or iatrogenic reaction to medications [6]. Additionally, OH affects patients with uncontrolled BP, especially those on three antihypertensive agents, with at least one being a diuretic or vasodilator [7]. Two studies have established possible links between vitamin D deficiency and a history of migraine with OH. A systematic meta-analysis by Ometto et al. of 3,646 participants revealed that patients with hypovitaminosis D had a higher prevalence of OH (odds ratio (OR) = 1.89, 95% confidence interval (CI) = 1.25-2.84; I = 68%) [8]. However, according to multiple studies, vitamin D deficiency is commonly a confounding variable, given its prevalence is high in the general population. A cohort from Blitshteyn and Cheshire of 323 patients with migraine revealed a 15% higher prevalence of syncope, with 12% having more than five incidences of syncope and an overall 20% higher incidence of OH [9].

Clinical manifestation and differentials

Clinically, patients with OH present with a constellation of symptoms, including blurred vision, dizziness, weakness, and fatigue. Some patients also complain of musculoskeletal somatic dysfunctions, such as shoulder or chest pain [1,5,6].

Differentials of OH can include, but are not limited to, (1) carotid sinus hypersensitivity: stimulation of carotid receptors, often trigged by tight collars. (2) Vasovagal syncope: often triggered by environmental stimuli such as visualization of bodily fluids, fear/emotions, or even due to physical exertion. (3) Postural orthostatic tachycardia: ANS dysautonomia with decreased BP and a rapid increase in HR. The complete mechanism remains unclear, and it affects more females and a younger population.

Clinical workup

A detailed history is vital to diagnosing patients with OH, along with a complete examination and thorough medication review. Once OH is suspected based on history and physical examination, the clinician should obtain the BP measurements when the patient is supine and again when the patient is upright for at least three minutes. Furthermore, the clinician should review vitals with EKG to rule out potential cardiac etiologies, such as arrhythmia.

Lab work should include a complete blood count with differentials to rule out any possible infectious etiology. A comprehensive metabolic panel is needed to evaluate intravascular volume status and electrolyte dyscrasia, which may cause underlying OH. To assess adrenal patency, the clinician may include specific hormonal tests in the workup, such as morning cortisol [5]. Urine studies can be used to determine the adequacy of salt intake. In rare cases, the clinician can use additional labs to screen for paraneoplastic antibodies in patients with syncope and autonomic failure [6].

If there is a concern for neurogenic OH, cerebral CT or even magnetic resonance imaging should be obtained to assess neurodegenerative disease(s) or to rule out stroke. An echocardiogram can be utilized in patients with known or suspected heart disease [5]. Tilt-table testing should be utilized in patients with suspected vasovagal syncope or suspected OH where orthostatic vitals are inconclusive. However, the tilt-table test should not be used to assess the efficacy of any medical therapy [10].

Treatment

Management of OH is geared toward symptomatic relief. First, understanding the instigating cause is paramount as simple medication or trigger avoidance can fix OH. Behavioral and lifestyle changes are equally crucial as dehydration might be the root cause of a patient’s OH. If a patient suffers from post-prandial syncope, they should adjust their feeding schedule to encourage smaller frequent meals, a decreased heavy carbohydrate diet, and limited alcohol consumption.

If a patient presents with neurogenic OH, the goal is to increase salt and water intake. A randomized trial by Schroeder et al. revealed that a 500 mL bolus of mineral water improved orthostatic tolerance by 5 ± 1 minute, p < 0.001 [11]. Chisholm et al. recommended targeting 6-10 g of salt daily in patients without the presence of hypertension with urine sodium >170 mmol/day, confirming adequate intake [7]. Other non-pharmacological options may include pressure compression devices which decrease venous pooling.

If non-pharmacologic options fail or are inadequate, medical therapy can assist with symptomatic relief. According to Lanier et al., patients with urine sodium <170 mmol per 24 hours should be given salt supplements of 0.5-1 g, up to t.i.d., with close monitoring of patients with underlying heart failure due to the risk of pulmonary edema [5]. A few first-line medications discussed below have shown efficacy in managing neurogenic OH symptoms.

Midodrine benefits patients with syncopal episodes caused by neurogenic OH [10]. Midodrine leads to the formation of desglymidodrine, the active metabolite, which is an alpha(1)-agonist which causes constriction of arteriolar→venous vasculature→increases TPR→ increases BP [12]. Dosage starts at 2.5 mg t.i.d. and can be titrated upward to a maximum of 10 mg t.i.d. [5]. The most significant contraindication would be in patients with underlying organic heart disease, acute renal disease, pheochromocytoma, urinary retention, or thyrotoxicosis [12]. Midodrine has been linked to supine hypertension, and excess alpha(1)-agonist activity can also lead to reflex bradycardia.

Fludrocortisone, part of the corticosteroid class, is a synthetic adrenocortical steroid with strong mineralocorticoid properties. The proposed mechanism of action is related to controlling the rate of protein synthesis, and, in small doses, it causes sodium retention with increased urine potassium excretion. High doses inhibit adrenal cortical secretion [13]. Increased sodium expands intravascular volume and has been shown to increase alpha-adrenergic sensitivity [6]. Contraindications include systemic fungal infections and prior adverse reactions. The usual dosage is 0.1-0.2 mg per os (PO). Review of Cochrane Neuromuscular Specialised Register where systemic review of three randomized crossover trials and ten studies evaluated fludrocortisone usage in 513 patients with OH. Results revealed no significant differences in hemodynamics in overall OH symptoms. However, there was a lack of information regarding long-term efficacy [14]. Thus, long-term and large-scale meta-analyses are required before clinicians can deduce proper conclusions.

Droxidopa is another medication that has been useful in patients with neurogenic OH [10]. It is a synthetic precursor for norepinephrine (NE). It converts to NE, which increases vasoconstriction in peripheral arteries greater than veins and leads to increased BP [15]. In a randomized trial by Kaufmann et al., 263 patients with symptomatic OH received droxidopa for seven to fourteen days. Droxidopa improved symptoms of symptomatic neurogenic OH and increased SBP [16]. The initial dosage starts at 100 mg t.i.d. and can be titrated to a maximum of 1,800 mg/day. Adverse effects include supine hypertension, nausea, dizziness, and headaches.

Pyridostigmine, an acetylcholinesterase (AChE) inhibitor, has also been shown to help patients with OH by enhancing sympathetic ganglionic transmission [5]. However, it has led to multiple gastrointestinal discomforts among patients and is not well tolerated [7]. Dosage starts at 30 mg two to three times and can be titrated to 60 mg t.i.d. [5]. A randomized trial studying pyridostigmine among 58 patients with neurogenic OH revealed pyridostigmine increased standing BP and improved DBP with no effect on supine hypertension [17]. However, a randomized trial of 87 adults with symptomatic OH treated with pyridostigmine, midodrine, or a combination of both revealed that midodrine was better at improving OH symptomatology [18].

Less frequently used second-line drugs include octreotide, alpha-sympathomimetics, erythropoietin for symptomatic anemic patients, and caffeine to treat OH [6,7]. Many other medications and supplements have been used, but the results are still inconclusive. A case series of seven patients revealed the benefit of coenzyme Q10 supplementation. On average, there was a mean SBP reduction of 7 mmHg versus 30 mm Hg without Q10 supplementation (p = 0.007) [19].

In-depth case discussion

Clinically, even with many pharmacologic options, managing OH is complicated, and each regimen is patient-specific. Even with a combination of midodrine, fludrocortisone, and Droxidopa, our patient still had OH symptomatology. Although Droxidopa has room to be titrated upward, all medications mentioned above have adverse effects, such as supine hypertension. On repeat follow-ups with the patient, it is worthwhile to note that the patient had lumbar pain, which he did not disclose during his initial hospitalization. There could be a possible vasovagal effect from the lumbar pain contributing to the patient’s OH symptomology. We further investigated this theory by having the patient perform Valsalva, which did drop BP; however, not to the same degree as when he stood up.

Furthermore, the interesting point is that even though our patient had positive orthostatic vitals, he did not have a compensatory increase in HR which would be expected based on the formula CO = HR × SVR (or TPR). Probably indicative of ANS reflex malfunction, particularly the SNS component. Additionally, when the patient was started on medications, he had supine hypertension, with both SBP and DBP elevated. Elevation of DBP meant the medical therapy had some efficacy as midodrine caused alpha(1)-agonist-induced vasoconstriction→increased afterload→increased TPR, and, hence, elevated pressure. Elevated SBP with elevated DBP could mean adequate fluid status as the body had volume to circulate the blood effectively. Interestingly enough, our patient had only two isolated episodes of supine hypertension episodes while tolerating the combination medications without any difficulty. This likely ties back to the initial point that his OH presentation was likely related to ANS dysautonomia.

Given the patient was at high fall risk and needed aggressive rehabilitation along with close monitoring of his vitals, it was appropriate to discharge the patient to an inpatient rehabilitation center. On discharge, the patient was given instructions to maintain a log of daily water intake, salt consumption, and daily BP readings and to record his BP whenever he felt dizzy or weak. A follow-up with the patient after six weeks revealed he had another ground-level fall. The fall was likely due to his underlying OH, and Droxidopa was titrated upward. The long-term efficacy of droxidopa for our patient warrants long-term observation, and, hopefully, this can lead to the resolution of his refractory OH.

## Conclusions

With the average life expectancy increasing, there is a possibility of an increasing number of elderly being affected with symptomatology consistent with OH. Therefore, it is essential to understand the pathophysiology of OH. Our OH review shows how challenging OH management can be even with multiple pharmaceutical and non-pharmaceutical options with adverse effects of supine hypertension to variable responses to each therapy. To properly gauge the efficacy of treatment, further longitudinal studies need to be conducted to manage and understand refractory OH.
